# Supplementary material for: Elevated CO2 Impacts on Plant–Pollinator Interactions: A Systematic Review and Free Air Carbon Enrichment Field Study
Source: Insects. 2021 Jun 1;12(6):512. doi: 10.3390/insects12060512 (PMC8227562; doi:10.3390/insects12060512)

Supplementary material

Table S1 – Systematic review articles by topic.

Topic	Articles
Flowering phenology	Bae and Sicher 2004; Bhattacharya et al. 1985; Bidart-Bouzat et al. 2004; Bloor et al. 2010; Bunce 2015; Carter et al. 1997; Case et al. 1998; Cleland et al. 2006; Curtis et al. 1994; Erhardt et al. 2005; Fabian et al. 2019; Farnsworth & Bazzaz 1995; Frick et al. 1994; Garbutt & Bazzaz 1984; Garbutt et al. 1990; Garruña-Hernández et al. 2012; He & Bazzaz 2003; He et al. 2005; Heinemann et al. 2006; Hicklenton & Jolliffe 1980; Hoover et al. 2012; Hovenden et al. 2009; Hovenden et al. 2008; Jablonski 1997; Johnston & Reekie 2008; Kinugasa et al. 2003; Kumar et al. 2014; Lake & Hughes 1999; Lee 2011; Leishman et al. 1999; McConnaughay et al. 1993; Musil et al. 1999; Osborne et al. 1997; Padhan et al. 2020; Posner 1971; Potvin & Strain 1985; Rämö et al. 2007; Rämö et al. 2006; Rathcke 1992; Reekie & Bazzaz 1991; Reekie et al. 1994; Reekie et al. 1997; Rogers et al. 2006; Rusterholz & Erhardt 1998; Seneweera et al. 1994; Sharma et al. 2020; Song et al. 2009; Van Der Kooij & De Kok 1996; Vanaja et al. 2015; Wagner et al. 2001; Wand et al. 1996; Ward & Strain 1997; Wolfe-Bellin et al. 2006; Woodin et al. 1992; Zhang & Lechowicz 1995.
Pollen	Albertine et al. 2014; El Kelish et al. 2014; Kim et al. 2018; Kobayasi et al. 2019; LaDeau & Clark 2006; Lake & Hughes 1999; López-Cubillos & Hughes 2016; Marshall et al. 2010; Prasad et al. 2011; Rogers et al. 2006; Silva et al. 2015; Singer et al. 2005; Wayne et al. 2002; Ziska & Caulfield 2000; Ziska et al. 2016.
Nectar	Dag & Eisikowitch 2000; Erhardt et al. 2005; Fabian et al. 2019; Hoover et al. 2012; Huber 1956; Lake & Hughes 1999; López-Cubillos & Hughes 2016; Osborne et al. 1997; Rathcke 1992; Rusterholz & Erhardt 1998.
Pollination	Dag & Eisikowitch 2000; Glenny et al. 2018; Hoover et al. 2012.

Table S2 - Residuals of ANOVA for treatment against First flowering day of year, mid flowering day of year and flowering duration.

Analysis of Variance Table					
Response: DOY_first					
	Df	Sum Sq	Mean Sq	F value	Pr(>F)
Treat	1	60.167	60.167	3.967	0.1172
Residuals	4	60.667	15.167		

Analysis of Variance Table					
Response: DOY_mid					
	Df	Sum Sq	Mean Sq	F value	Pr(>F)
Treat	1	60.167	60.167	12.893	0.02295*
Residuals	4	18.667	4.667		

Analysis of Variance Table					
Response: DOY_duration					
	Df	Sum Sq	Mean Sq	F value	Pr(>F)
Treat	1	0.167	0.1667	0.0091	0.9286
Residuals	4	73.333	18.3333		

Table S3 – Correlation matrix of flowering time and environmental variables.

	DOY_first	DOY_duration	DOY_median	Temp	Moist	LUX
DOY_first	1	-0.6738088	0.9409185	-0.3620968	0.7116293	0.675191
DOY_duration	-0.6738088	1	-0.3875461	0.4885743	-0.5887001	-0.4963
DOY_median	0.9409185	-0.3875461	1	-0.2287862	0.5860794	0.585017
Temp	-0.3620968	0.4885743	-0.2287862	1	-0.3770705	-0.46252

Moist	0.7116293	-0.5887001	0.5860794	-0.3770705	1	0.978308
LUX	0.6751914	-0.4962997	0.5850172	-0.4625195	0.9783081	1

Table S4 – All species/species group recorded visiting bluebells during surveys.

**Order**	**Family**	**Species/Species group**	**Percent of total visits**
Diptera	Syrphidae	Platycheirus spp.	35
Hymenoptera	Apidae	Bombus pratorum	17
Diptera	Syrphidae	Rhingia campestris	15
Diptera	Syrphidae	Syrphus spp.	10
Hymenoptera	Apidae	Bombus lapidarius	8
Diptera	Syrphidae	Melanostoma scalare	8
Hymenoptera	Ichneumonidae	Ichneumonidae spp.	8
Coleoptera	Elateridae	Athous sp.	8
Diptera	Bibionidae	Bibio sp.	6
Hymenoptera	Apidae	Bombus pascourum	4
Diptera	Syrphidae	Baccha elongata	4
Diptera	Syrphidae	Lecozona lucorum	4
Hymenoptera	Apidae	Bombus terrestris	2
Coleoptera	Cantharidae	Cantharis decipiens	2
Coleoptera	Coccinellidae	Tytthaspis sedecimpunctata	2
Mecoptera	Panorpidae	Panorpa sp.	2
Lepidoptera	Pieridae	Pieris rapae	2

Figure S1 – Mean monthly soil temperature for 3 years proceeding observed flowering period.

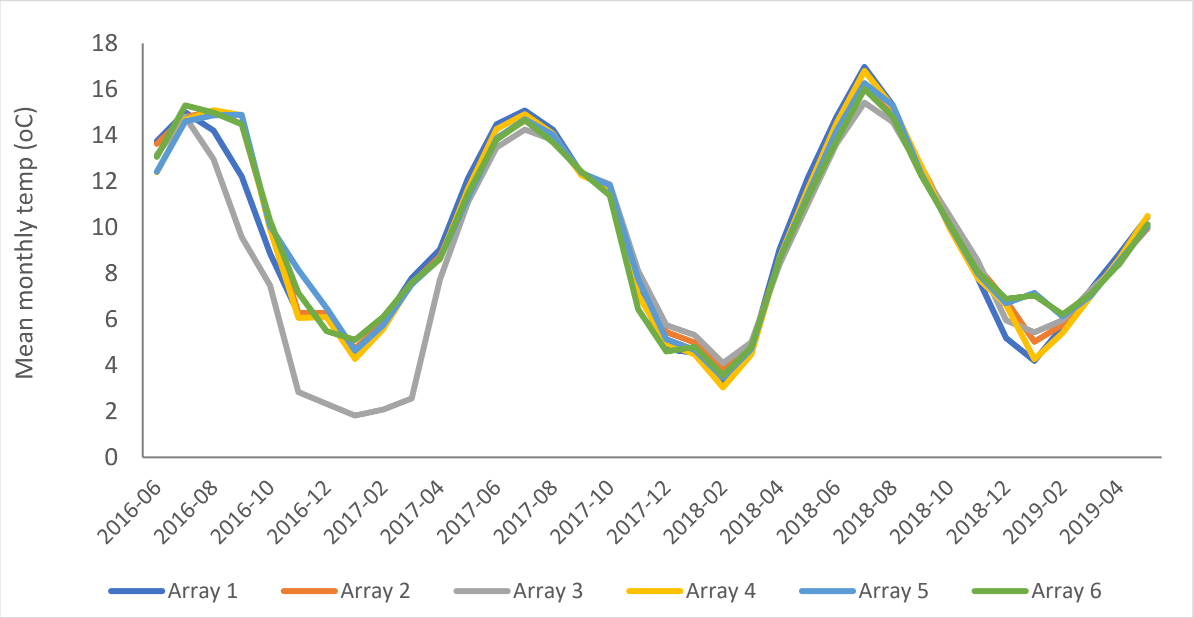

Figure S2 – Mean monthly soil moisture (m3/m3) for each array for 3 years proceeding observed flowering period.

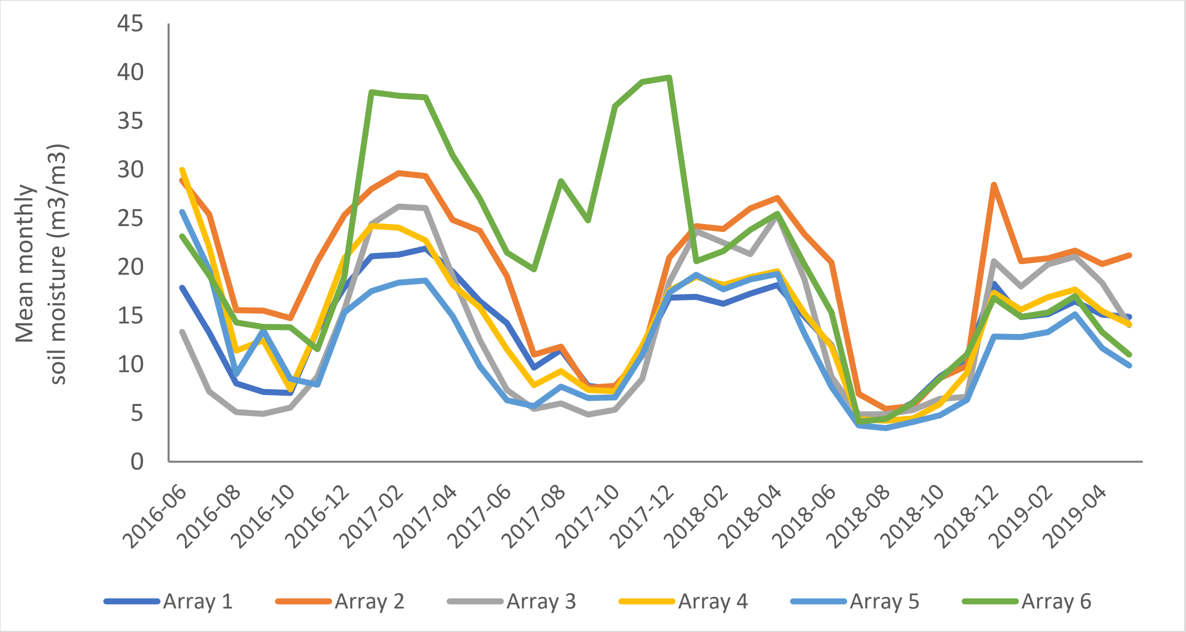

Supplement: Supplementary file 1 [file insects-12-00512-s001.zip › insects-1160262-supplementary-2nd proofreading done/Supplementary material.pdf]
